# Foliar-applied honokiol exhibits basipetal translocation, offering a strategy for root disease management in precision agriculture systems

**DOI:** 10.3389/fpls.2026.1846319

**Published:** 2026-05-21

**Authors:** Zhangguang Cao, Guoyu Wei, Amir Khan, Anlong Hu, Zhenxiang Guo

**Affiliations:** Department of Plant Protection, College of Agriculture, Guizhou University, Guiyang, Guizhou, China

**Keywords:** basipetal translocation, crop improvement, in silico analysis, natural pesticides, root rot disease, sustainable agriculture practice

## Abstract

**Introduction:**

Root diseases in crops are increasingly becoming the most difficult to control due to the challenge of fungicides reaching infection sites. Honokiol, a natural fungicidal product derived from *Magnolia officinalis*, is primarily biosynthesized in leaves and subsequently translocated to roots to resist pathogen infection.

**Methods:**

In this study, honokiol applied to the leaves of *Polygonatum cyrtonema* Hua (PCH) could be transported downward to the roots and exhibited a control effect against root rot disease.

**Results:**

Subsequent research showed that concentration, temperature, pH, energy inhibitors (DNP, CCCP) and other agents (AgNO_3_, BaCl_2_, CaCl_2_) significantly affect basipetal translocation of honokiol, suggesting that its translocation from leaves to roots is mediated by transport proteins. The distribution of honokiol in various parts of PCH was found as leaf > stem > root, indicating that honokiol was absorbed by the leaves and transferred to the roots through phloem. Translocation factor_root/stem_ value of honokiol in PCH was more than 1, indicating that honokiol was easy to transfer to the root. Subsequently, four potential transporter proteins, ABCB11, ABCG24, TIP1–2 and PIP1-3, were screened from *Arabidopsis thaliana* for molecular docking analysis, indicating a significant binding affinity between honokiol and transporter proteins having a binding energy of −12.83 kcal mol^-1^, −10.70 kcal mol^-1^, −9.38 kcal mol^-1^ and −10.67 kcal mol^-1^, respectively.

**Discussion:**

Foliar-applied honokiol exhibited pronounced basipetal translocation, efficiently moving from leaves to root tissues where root rot pathogens reside. This systemic redistribution enables targeted delivery of the antifungal compound honokiol to the infection site without direct soil application, minimizing environmental exposure.

## 1 Introduction

Diseases continuously attack crop plants, leading to decreased yields and diminished nutritional and organoleptic value (Agrios, 2004). PCH is a perennial herb that has been widely used in Chinese herbal medicine. Its rhizomes are rich in compounds such as saponins, nicotinic acid, sugars, quinones, and amino acids and demonstrate significant efficacy in lowering blood sugar, anti-tumor effects, improving immunity, and delayed ageing (Tang et al., 2023; Liu et al., 2022a). However, in the PCH planting process, root rot, a common soil-borne disease, seriously affects the growth, quality, and yield of plants (Mei et al., 2024; Thomidis et al., 2009). A complex of soil-borne pathogens, viz., *Fusarium* spp., *Pythium* spp., and *Rhizoctonia solani* are responsible for root rot disease (Liu et al., 2022b; Zhang et al., 2023). At the moment, chemical fungicides like tebuconazole, carbendazim, and myclobutanil are often used to control root rot in PCH by watering the roots (Zheng et al., 2024; Wang et al., 2023). However, this method has two major drawbacks. First, due to soil isolation and adsorption, only a scant amount of fungicide reaches the target disease sites, resulting in unsatisfactory control efficacy. Second, the extensive use of chemical pesticides leads to product and environmental contamination. To tackle these challenges, research should concentrate on two areas: first, the identification of highly effective and safe natural product fungicides; second, the development of root-targeted translocation methods for fungicides. This can be realized through foliar application, enabling accumulation in the roots and thereby achieving precise disease control.

Many pesticidal active compounds are derived from natural plant products, particularly in herbs including sage, garlic, oregano, and thyme (Jamiołkowska, 2013; Kawka et al., 2017). They have a minimal adverse effect on the environment, limit the growth of pathogenic fungus, and inhibit the generation of mycotoxins. The *Magnolia* species is the source of the lignan known as honokiol (3,5-di-2-propenyl-1,1-biphenyl-2,4-diol) (Prasad and Katiyar, 2016). Honokiol has high antifungal activity and is an excellent candidate for sustainable and commercially available fungicides that are harmless for humans and animals (Oufensou et al., 2019). A large number of studies have suggested that the main mechanisms for the conduction of exogenous compounds in the phloem of plants are related to the oil-water partition coefficient LogP and acid ionization constant pKa (Cai et al., 2022; Li et al., 2018). The most widely accepted theories are the weak acid theory and intermediate permeability theory, which suggest that when pKa is between 3 and 6 and logP is between −0.5 and 4, xenobiotics may have phloem conductivity (Kleier, 1988). However, this representation cannot explain the phloem mobility of some pesticides that do not conform to it, such as glyphosate. The broad bean’s absorption of the herbicide glyphosate demonstrated that a phosphate transporter of the plasma membrane mediated the uptake of glyphosate (Denis and Delrot, 1993).

Currently, most systemic pesticides are transported upward through the xylem (Zhang et al., 2023). For a long time, researchers have been searching for endogenous conductive pesticides that can be transported through the phloem of plants. This way, stem and leaf spray can be used to control and treat root and vascular diseases, and untreated plant tissues and organs can be protected from chemical pollution. This also helps avoid the effects of soil factors on pesticides and maximizes their effectiveness. However, until now, no such pesticides have been developed except for the herbicide glyphosate. Some leaf-applied fungicides and insecticides can be transmitted to the roots, but the proportion of transmission is low, which cannot meet the requirements of control concentration; therefore, alternative methods or formulations, such as increasing the concentration of active ingredients or using adjuvants to improve absorption, may be necessary to enhance their effectiveness in pest control. Honokiol is synthesized in the leaves of *M. officinalis* and transported downward through the plasma membrane and sieve tube and finally accumulated in the root bark of *M. officinalis* to resist pathogen infection (Shi et al., 2017).

Molecular docking is a computational technique that predicts the preferred orientation and conformation of honokiol when it is bound to a receptor (transporter proteins). A popular *in silico* method for analyzing molecular relationships at the structural level is molecular docking analysis, which forecasts the binding orientation and affinity between a ligand and its receptor, usually a protein. Molecular docking is being employed more and more in agricultural research to investigate the action mechanisms of phytochemicals and nanoparticles, pesticide-target specificity, and plant–pathogen interactions (Khan et al., 2020). Protein–ligand interactions can be studied at the atomic level using molecular dynamics simulations, which also make it possible to characterize the complexes’ stability under physiological settings, conformational changes, and binding kinetics (Salo-Ahen et al., 2020). This study examines the potential for foliar-applied honokiol to translocate to the roots in various plant species, evaluates its effectiveness in managing root diseases, and conducts an initial investigation into its transport mechanisms. This research is crucial for the advancement of new natural fungicides aimed at effectively preventing vascular bundle and soil-borne diseases while minimizing interior plant and soil pollution.

## 2 Materials and methods

### 2.1 Chemicals and biological materials

Honokiol (98%) was purchased from Aladdin Reagent Co., Ltd (Shanghai, China); its solution was prepared by first dissolving it in dimethyl sulfoxide, then diluting it to different concentrations with a 0.1% Tween 80 solution. Acetonitrile [high-performance liquid chromatography (HPLC) grade] and methanol (HPLC grade) were obtained from German Merck Reagent Co., Ltd (New York, USA). PCH was grown in plastic pots (one plant per pot; the temperature was 25 °C during the day and 15 °C at night). Two-year-old plants with 8 leaves were selected for the experiment. The root rot pathogen *Fusarium commune* was isolated and preserved in the laboratory by our research group.

### 2.2 Control root rot of PCH by foliar applied honokiol

Three treatments were set up: foliar applied honokiol, honokiol root drenching and control. A *F. commune* spore suspension of 1 × 10^6^ conidia/mL was used for artificial inoculation to induce root rot in PCH. Protective and curative efficacy are measured by inoculation 24 h after application and 24 h before application, respectively. Honokiol at concentrations of 25, 50, and 100 mg L^-1^ was applied 3 times, respectively, at 7-day intervals, with 50 mL per plant per time. Three repetitions of each treatment were used in the experiment. The pot opening in the foliar spray treatment was covered with aluminum foil to prevent solution droplets from falling into the soil. Control efficacy was assessed 7 days after the final application using the following formula. The grading criteria of disease severity were shown in **Table 1**.

**Table 1:** Grading criteria of disease classification standard of infected root of PCH.

Disease grade	Symptoms
0	Healthy
1	Ground stems and leaves less than 1/2 (including 1/2) wilted, the root appearance is normal
3	Above ground stem and leaves more than 1/2 (not including 1/2) wilted, root and stem vascular bundle brown, not yet lignified.
5	Part of the root began to rot, and the rotten part accounted for less than 30% of the volume of the root and stem (including 30%)
7	All the stems and leaves on the ground are withered, and the rotten part accounts for more than 30% of the volume of the rhizomes (excluding 30%)
9	The stems and leaves in the ground are dead, and the roots are all rotten


$$\text{Disease index}=\frac{\sum (\text{Number of diseased plants in each disease grade}\times \text{disease grade})}{(\text{Total plants examined}\times 9)\times 100}$$

$$\text{Control efficacy}\;(\%)=\frac{\text{Disease index of control − Disease index of honokiol treatments}}{\text{Disease index of control}\times 100}$$

### 2.3 Determination of uptake and translocation characteristics and mechanism of foliar applied honokiol in PCH

**Figure 1:**
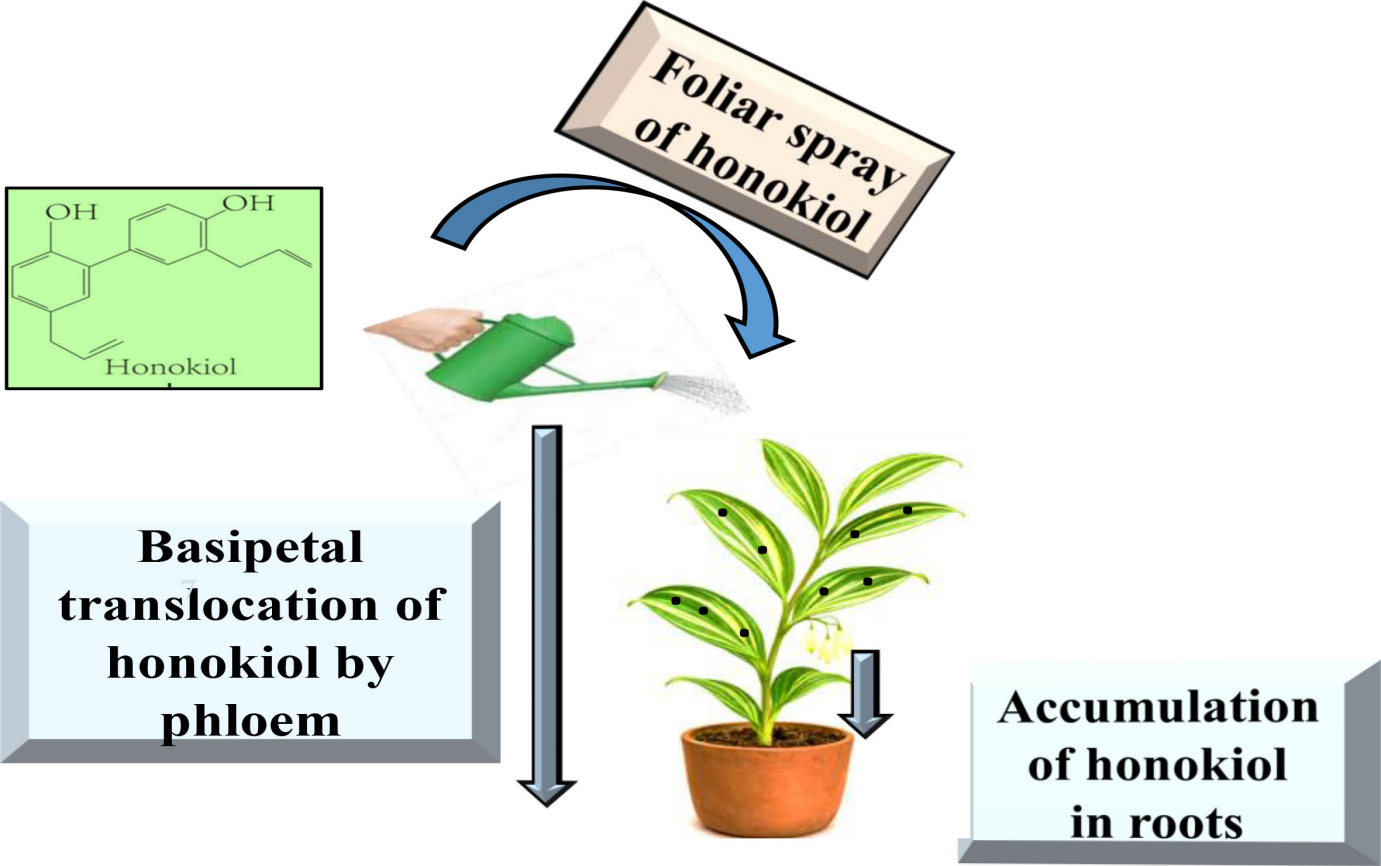
Schematic diagram of the leaves of PCH treated with honokiol showing basipetal translocation.

To elucidate the distribution of honokiol in the plant, a honokiol solution of 100 μg mL^-1^ was applied to the middle four leaves of PCH (**Figure 1**, leaves 3–6). After 24 h, various tissue sections were collected. To assess translocation factor and enrichment factor, the sprayed leaf, lower stems, and roots were collected at 12, 24, 48, 72, 96, and 120 h after foliar spraying of honokiol. To elucidate the effect of concentration on uptake and translocation, 0.093, 0.187, 0.375, 0.75, 1.5, and 3 mmol L^-1^ honokiol solutions were sprayed, respectively, and the lower stems were collected after 24 h. To investigate the effect of temperature on uptake and translocation, honokiol was sprayed and cultured at 4°C and 24°C. The lower stems were collected after 12, 24, and 48 h. To clarify the effect of pH on uptake and translocation, the pH of honokiol solution was adjusted to a series of solutions ranging from 4 to 9 with 1 mol L^-1^ sodium hydroxide and citric acid, and the prepared solution was sprayed, and the roots, lower stems, and sprayed leaves were collected after 24 h.

Energy inhibitors carbonyl cyanide chlorophenylhydrazone (CCCP) and dinitrophenol (DNP) were used to investigate whether the uptake process is energy-dependent (Zhang et al., 2022). The 0.1, 0.5, and 1 mmol L^-1^ DNP and 0.1, 0.5, and 1 mmol L^-1^ CCCP were applied 1 h before honokiol spraying, respectively. Channel inhibitors were employed to preliminarily determine the pathway for honokiol entry into the plant. Ten μmol L^-1^ of AgNO_3_ (aquaporin inhibitor), 50 μmol L^-1^ of 4, 4′-diisothiocyano-2, 2′-stilbenedisulfonate (DIDS, anion channel inhibitor), 5 mmol L^-1^ BaCl_2_ (potassium channel inhibitor) and 5 mmol L^-1^ CaCl_2_ (cation channel inhibitors) were sprayed with honokiol, respectively. All treatments were applied with honokiol at 0.375 mmol L^-1^, a volume of 50 mL per replicate, and three replicates per treatment, using water spray as the control, unless otherwise specified.

### 2.4 Sample preparation and purification for ultra-performance liquid chromatography and tandem mass spectrometry (UPLC-MS/MS) analysis

Each sample (leaves, stem and root) was first washed with water and then washed with 10% methanol to remove honokiol from the surface. Weigh 2.0 g of each sample and add the appropriate amount of liquid nitrogen to cover the plant tissue and then grind it. The powdered sample was transferred to a 50 mL polytetrafluoroethylene (PTFE) centrifuge tube; 10 mL of extraction agent (methanol) was added and vortexed for 5 min, 1 g NaCl was added and vortexed again for 5 min, and centrifuged at 4000 rpm for 5 min. Then 1.2 mL supernatant was taken into a 2 mL centrifuge tube containing 150 mg anhydrous MgSO_4_ and a purification agent (10 mg GCB + 50 mg PSA for leaves and stems, 50 mg PSA for roots) and vortexed for 1 min. After centrifugation at 12000 rpm for 5 min, about 1 mL of supernatant was taken and subjected to a 0.22 μm PES membrane for detection and analysis by UPLC-MS/MS. Three replicates were examined for each treatment.

### 2.5 UPLC-MS/MS analysis of honokiol

Analysis of honokiol was performed using the UPLC-MS/MS analysis. An Agilent 1,290 series UPLC equipped with a binary pump, auto-plate sampler, column, and Agilent 6,470 triple quadrupole mass spectrometer (MS/MS; Agilent Technologies, United States) was used for the analysis of honokiol. The chromatography was performed on an Agilent Eclipse plus C18 column (4.6 × 100 mm, 3.5 μm diameter). The following UPLC settings were used: Flow rate, 0.3 mL min^-1^; column temperature: 30 °C; injection volume, 5 μL. Mobile phase A consisted of a 0.1% formic acid aqueous solution, and mobile phase B consisted of acetonitrile; running time was 6 min. The mass spectrometer was operated in negative electrospray ionization (ESI) mode using multiple reaction monitoring (MRM). The MS parameters of the target compound are as follows: Sheath gas temperature and sheath gas flow: 250°C and 11.0 L/min; nozzle voltage, 500 V; capillary voltage, 3500 V; atomizing gas pressure, 45 psi; gas temperature and gas flow, 300°C and 5 L/min; fragmentation voltage, 120 V; quantitative ion pair: m/z 265.1/224; collision energy, 25 eV; Qualitative ion pair: m/z 265.1/247, collision energy 35 eV. The gas, atomizing gas, collision gas and sheath gas are all high-purity nitrogen. Recovery studies were developed at three spiking levels of 0.1, 0.5, and 1 mg kg^-1^, and the individual mean recovery rates for honokiol were ~83.37%–95.43% (**Supplementary Appendix Table 1**). The standard curve is y = 443068x – 3890.2 (R² = 0.9943) (**Supplementary Appendix Figure 1**).

### 2.6 Molecular docking methodology

Due to the limited data on proteins in PCH, we performed molecular docking of honokiol with several key *A. thaliana* membrane transporter proteins to preliminarily screen for potential transporters for honokiol (Khan et al., 2025). Three-dimensional crystal structures of the potential proteins were retrieved from the Protein Data Bank (PDB). Prior to docking, all protein structures were prepared by removing crystallographic water molecules, co-crystallized ligands, and non-essential heteroatoms, followed by the addition of polar hydrogen and assignment of Kollman charges using AutoDock Tools (ADT). The ligand molecule, honokiol, was retrieved from the PubChem database and prepared by geometry optimization and addition of Gasteiger charges. Torsional degrees of freedom were set to allow full conformational flexibility during docking, while the receptor proteins were kept rigid. Docking simulations were performed using AutoDock 4.2 with the Lamarckian Genetic Algorithm (LGA) as the search strategy. For each target, a grid box of 120 × 120 × 120 points with a spacing of 0.375 Å was centered on the putative active site or translocation channel to ensure that the ligand had sufficient conformational space to explore possible binding modes. A total of 100 independent docking runs were performed per target protein to enhance the reliability of the predicted binding conformations (Jameel et al., 2025). The docking solutions were ranked based on the predicted binding free energy (ΔG, kcal mol^-1^), with the most favorable conformation selected for detailed interaction analysis. The docked complexes were further visualized and analyzed for hydrogen bonding, hydrophobic interactions, and π-related interactions using Discovery Studio Visualizer (Biovia) (Amir et al., 2024). Key interacting residues, bond distances, and binding orientations were carefully documented to understand the structural basis of honokiol’s affinity towards these transport proteins.

### 2.7 Data analysis

The data were analyzed using Microsoft Excel 2021 (Microsoft Inc., Redmond, MA, USA), and plots were generated using OriginPro 2021 (OriginLab Corporation, Northampton, MA, USA). One-way analysis of variance (ANOVA) was conducted using DPS v16.0 (Ruifeng Information Technology Co., Ltd., Zhejiang, China), and Tukey’s multi-range test was used to determine statistical significance at P < 0.05. The temperature coefficient (Equation 1) (Q_10_, the factor by which the reaction rate increases when the temperature is raised by ten degrees) equation is (Mundim et al., 2020):

(1) $$Q_{10}={\left[\frac{{\text{K}}_{2}}{{\text{K}}_{1}}\right]}^{\begin{array}{c} 10\\ T_{2}-T_{1} \end{array}}$$

T_2_ - Higher temperature; T_1_ - Lower temperature; K_2_ - Uptake at higher temperature; K_1_ - Uptake at lower temperature.

The root bio-concentration factor (RCF) (Equation 2), stem bio-concentration factor (SCF) (Equation 3), leaf bio-concentration factor (LCF) and translocation factor (TF) were calculated by the following Equation 4 (El-Mahrouk et al., 2024; Xie et al., 2023; Tao et al., 2021) (Equations 5, 6):

(2) $$\text{RCF}={\text{C}}_{\text{root}}/{\text{C}}_{\text{solution}}$$

(3) $$\text{SCF}={\text{C}}_{\text{stem}}/{\text{C}}_{\text{solution}}$$

(4) $$\text{LCF}={\text{C}}_{\text{leaf}}/{\text{C}}_{\text{solution}}$$

(5) $$\text{TF}={\text{C}}_{\text{stem}}/{\text{C}}_{\text{leaf}}$$

(6) $$\text{TF}={\text{C}}_{\text{root}}/{\text{C}}_{\text{stemt}}$$

Where, C_root_, C_stem_, C_leaf_ and C_solution_ represent the concentrations of honokiol in root (mg kg^−1^), stem (mg kg^−1^), and leaf (mg kg^−1^) of PCH and solution concentration prepared with honokiol (mmol L^-1^), respectively.

## 3 Results and discussion

### 3.1 Efficacy of honokiol foliar spray against root rot disease in PCH

The results showed that the efficacy of foliar spraying and soil drenching was almost identical (**Table 2**). Increasing the concentration from 25 to 100 mg L^-1^ significantly boosted the efficacy of honokiol. At the 100 mg L^-1^ of honokiol, protective and curative efficacy was found superior by the foliar application and soil drenching. The protective efficacy was found to be higher than its curability. The protective efficacy of honokiol on root rot disease was 41.36-82.74%, and the curative efficacy was 41.34-75.85% in foliar spray treatment. But in root-drenching treatment, the protective efficacy of honokiol was 43.68-85.04%, and the curative efficacy was 43.67-78.10%. The results showed that honokiol’s efficacy on root rot disease was similar between foliar spraying and root drenching treatments. Natural pesticides capable of translocating within the phloem of plant tissue offer significant benefits in managing root diseases, vascular bundle disorders, concealed drill wire issues, and subterranean pests that are challenging to detect in the initial stages of infection, while also enhancing targeting and utilization efficiency (Kawka et al., 2017; Zheng et al., 2020).

**Table 2:** Efficacy of honokiol foliar spray against root rot disease in PCH.

Application type	Mode of action	Concentration (mg L^-1^)	Disease index mean ± SD	Efficacy (%) mean ± SD
Foliar spray	Protective	25	20.99^b^ ± 2.46	41.36^e^ ± 6.88
		50	13.58^c^ ± 2.47	62.06^d^ ± 6.90
		100	6.17^f^ ± 1.23	82.74^a^ ± 3.44
	Curative	25	20.99^b^ ± 2.46	41.34^e^ ± 6.89
		50	13.58^c^ ± 2.47	62.05^d^ ± 6.90
		100	8.64^e^ ± 1.23	75.85^b^ ± 3.44
Root drench	Protective	25	20.16^b^ ± 0.71	43.68^e^ ± 1.98
		50	11.94^d^ ± 0.70	66.64^c^ ± 1.96
		100	5.35^f^ ± 0.70	85.04^a ±^1.97
	Curative	25	20.16^b^ ± 0.70	43.67^e^ ± 1.97
		50	12.75^cd^ ± 0.71	64.36^cd ±^1.98
		100	7.83^e^ ± 0.70	78.10^b^ ± 1.97
Control		–	35.80^a^ ± 2.47	–

The mean values sharing the same letters within a column are not significantly different from each other, while the mean values with different letters are significantly different (P<0.05).

### 3.2 Distribution of honokiol in PCH after foliar spray for 24 h

**Figure 2:**
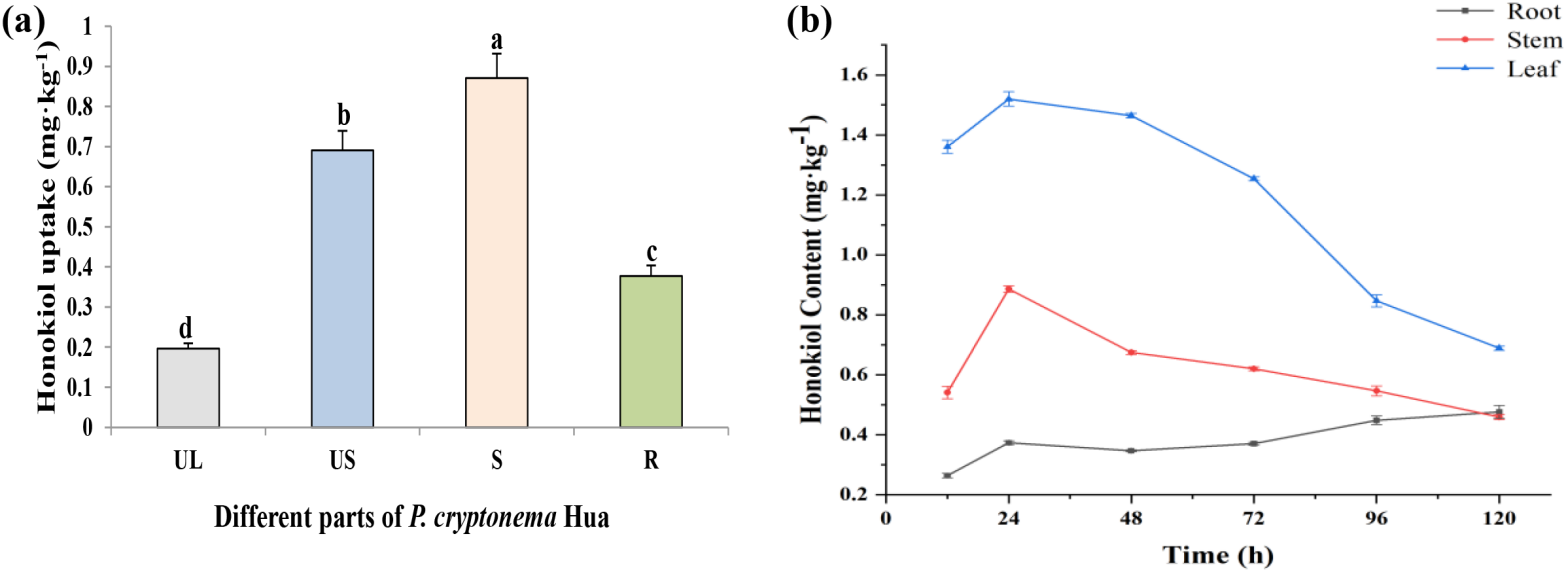
**(a)** Distribution of honokiol in different parts of PCH following treatment applied to middle leaves. The bars sharing the same letters are not significantly different from each other, while the bars with different letters are significantly different (P<0.05). **(b)** Time course analysis of honokiol uptake and distribution after 24-120 h of application. (UL: Upper leaves, US: Upper stem, S: Stem, R: Root, LCF: Leaf enrichment, SCF: Stem enrichment, RCF: Root enrichment).

As shown in **Figure 2A**, honokiol was detected in upper leaves, upper stems, lower stems and roots of PCH after the foliar application of 0.375 mmol L^-1^ honokiol for 24 h, indicating that honokiol can be transported bidirectionally in PCH. The content of honokiol in the lower stem was the highest and in the upper leaf was the lowest, indicating that the basipetal translocation of honokiol is predominant. Different pesticides have different directions of propagation (upwards, downwards, or bi-directionally) in the plant, and their modes of conduction, speed, and biological concentration are different when treating different plant organs (Sun et al., 2023). For instance, the contents of imidacloprid sprayed were different in cotton flowers, leaves, nectary tissues and pollens, being highest in leaves, but the imidacloprid residue was below the limit of quantification in flowers, nectariferous tissue and pollen from seed-treated (Brar et al., 2022).

### 3.3 Dynamic distribution and transport characteristics of foliar applied honokiol in PCH

The variation in honokiol content in various parts (root, stem, and leaf) of PCH over different time intervals (24, 48, 72, 96, and 120 h) was shown in **Figure 2B**. It was found that the content of honokiol detected in leaves was significantly higher than that in roots and stems at each time point. After spraying with honokiol on the leaves of PCH seedlings for 12 h, the content of honokiol detected in the roots of PCH seedlings was 0.2642 ± 0.0073 mg kg^-1^. The duration of honokiol exposure increased from 12 to 120 h in the leaves; the amount of honokiol rose rapidly to 0.3737 ± 0.0061 mg kg^-1^ at 24 h, then decreased slowly before rising again, and at 120 h, the content measured was 0.4764 ± 0.0215 mg kg^-1^. The content of honokiol in the stem was higher than in the root and increased from 12 to 24 h and decreased from 24 to 120 h. The concentration in the leaves increased to a peak of 1.5198 ± 0.024 mg kg^-1^ before continuing to decrease, which may be due to the uptake of honokiol by the roots and its partial degradation.

From the above results, the amount distribution of honokiol in various parts of PCH was found as leaf > stem > root, indicating that honokiol was absorbed by the leaves and transferred to the roots through the phloem. The detection of the honokiol amount in stems and roots indicates that it has some translocation ability (Fan et al., 2021). It was found that honokiol could be detected in the roots of PCH after 12 h of leaf spraying, and the content was 0.2642 ± 0.0073 mg kg^−1^. From 12 h to 24 h, the concentration of honokiol detected in the roots of PCH increased gradually and increased rapidly to 0.3737 ± 0.0061 mg kg^-1^ at 24 h, then decreased slowly and then increased rapidly. At 120 h, the content was 0.4764 ± 0.0215 mg kg^−1^. The concentration increased in the leaves to the highest point of 1.5198 ± 0.024 mg kg^−1^ and then continued to decrease. The detection of honokiol content in the stem and root showed that honokiol was absorbed by leaves and transferred to the stem and root through phloem, which had a certain transport capacity.

**Figure 3:**
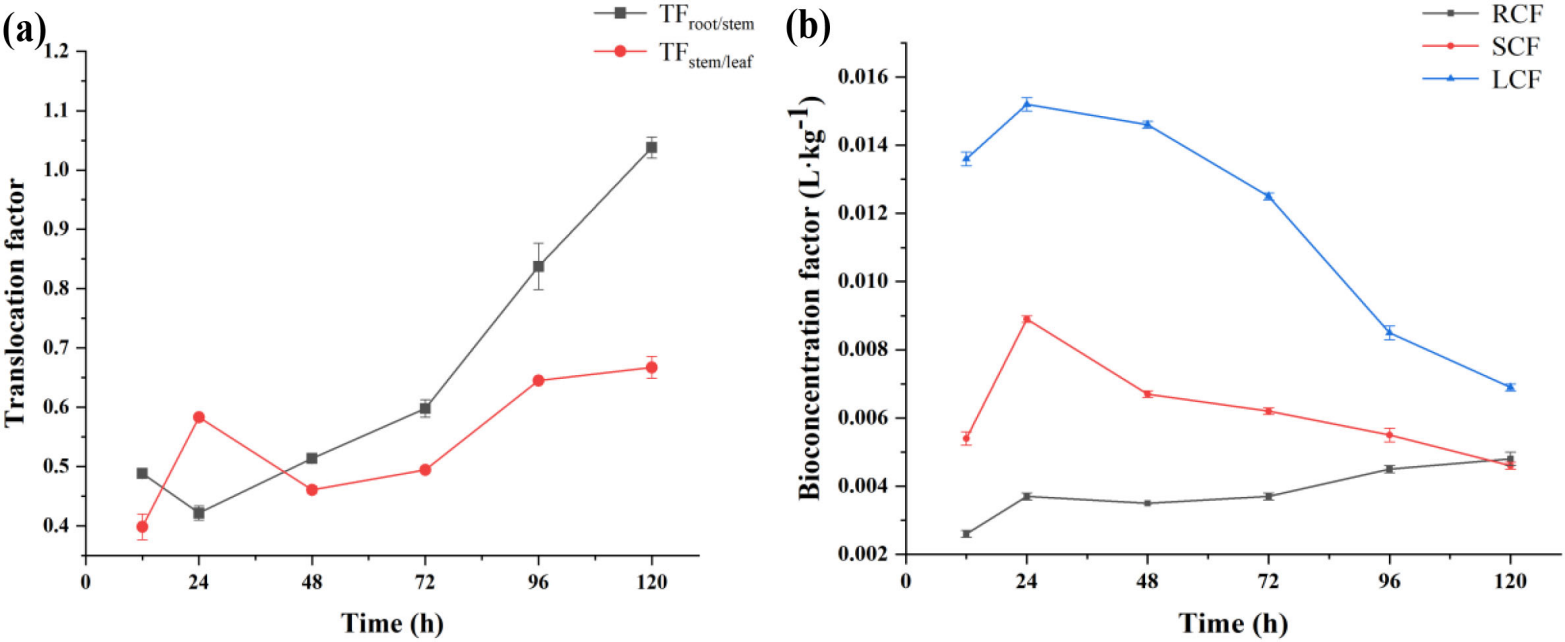
**(A)** Time course analysis of honokiol transport in PCH seedlings after 24–120 h of application. **(B)** Bio-enrichment and accumulation of honokiol in different parts of PCH after 24–120 h of application. (LCF, Leaf enrichment; SCF, Stem enrichment; RCF, Root enrichment).

Translocation factor (TF) was selected to evaluate the transport degree of honokiol from leaves to stem and root (**Figure 3A**). TF_root/stem_ showed a decreasing trend 12 to 24 h after application and an increasing trend after 24 h, with an average value of 0.65. However, the TF_stem/leaf_ exhibited an increasing trend from 12 to 24 h, followed by a decreasing trend from 24 to 120 h, and then a slow increasing trend, with an average value of 0.54. At 12–120 h after application, TF_root/stem_ was greater than TF_stem/leaf_, and the transport intensity from stem to root increased continuously at 24 h, but the transport intensity from leaf to stem did not change much, indicating that the transport of honokiol from stem to root was easier than that from leaf to stem. The TF_root/stem_ value of honokiol in PCH was more than 1, indicating that honokiol was easy to transfer to the root.

The bio-enrichment ability of honokiol in PCH was studied by analyzing the dynamic change in the bio-enrichment factor of honokiol of PCH plants with time. The LCF of honokiol in PCH increased rapidly before 24 h and then decreased from 24 h to 120 h, as shown in **Figure 3B**, and the value at 120 h was found to be 0.0069 ± 0.0001 L kg^-1^. The SCF value increases rapidly first and then decreases slowly; the value of 24 h was 0.0089 ± 0.0001 L kg^-1^. The RCF rose rapidly first and then slowly increased, and the value of 120 h was 0.0048 ± 0.0002 L kg^-1^. The results showed that LCF was higher than SCF and RCF, indicating that the biological enrichment capacity of honokiol in PCH leaves was much higher than that in stems and roots. According to the findings, honokiol was easily absorbed by the leaves of the plant and then moved upward, which led to a relative enrichment in the roots (Tao et al., 2021).

### 3.4 Concentrations dependence uptake of foliar applied honokiol in PCH

**Figure 4:**
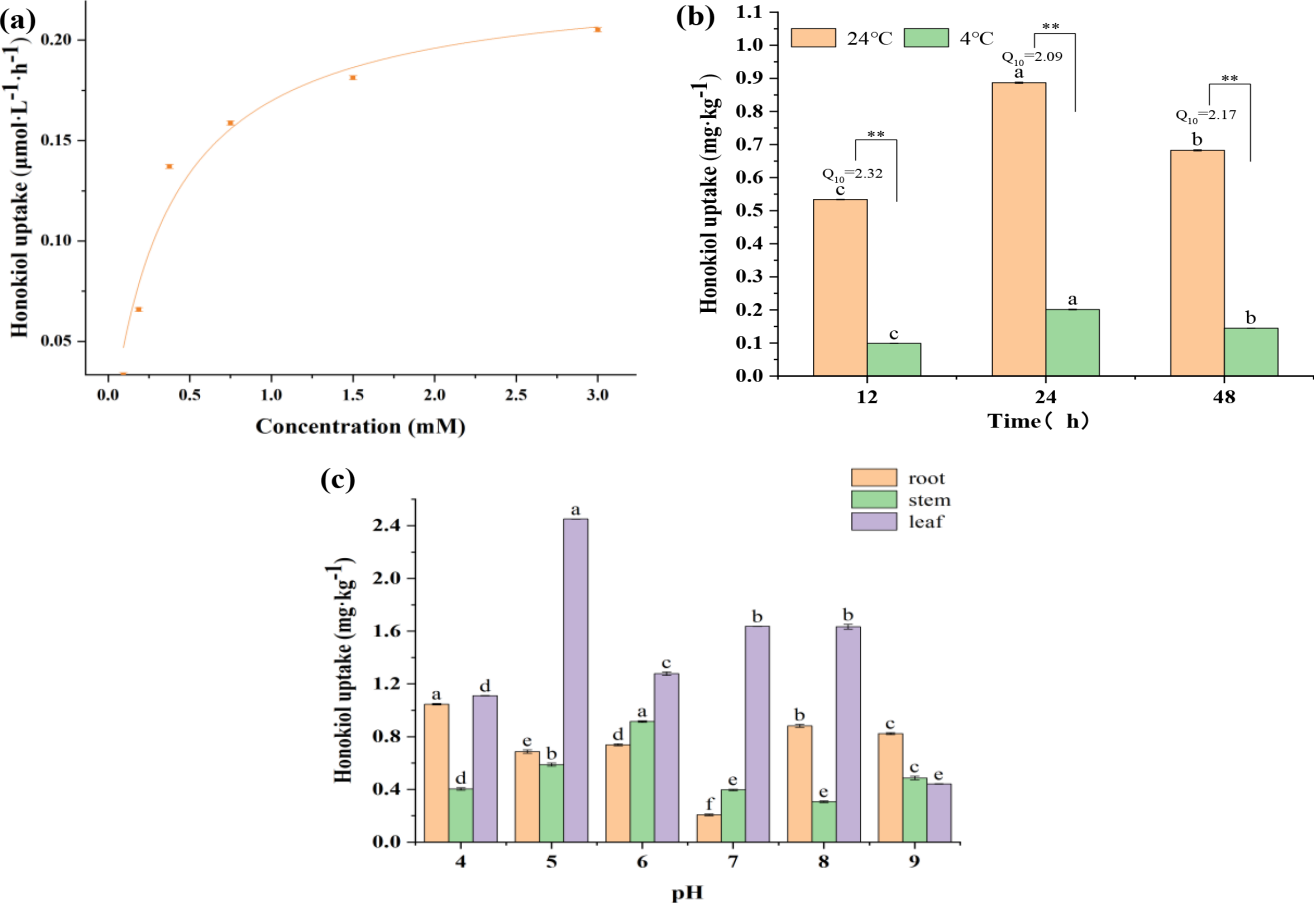
**(a)** Effect of varying concentrations on the uptake efficiency of foliar-applied honokiol in PCH. **(b)** Effect of low and ambient temperatures (4°C and 24°C) on the uptake of honokiol in PCH. The asterisk indicates *p < 0.05*, which represents a highly significant level. **(c)** Effect of varying pH on the uptake of honokiol in PCH. The bars sharing the same letters are not significantly different from each other, while the bars with different letters are significantly different (P<0.05).

As shown in **Figure 4A**, the results of UPLC-MS/MS analysis showed that honokiol was detected in the stems of PCH after 24 h of treatment with different concentrations. After the application of different concentrations of honokiol, the uptake amount of honokiol was found significantly different, indicating that the uptake of honokiol was concentration-dependent. At low concentrations (0.093-0.375 mmol L^-1^), the uptake of honokiol content in the stem of PCH showed a linear relationship. The uptake of honokiol by PCH seedlings was found saturated, approaching saturation at 0.375 mmol L^-1^. At high concentrations (0.375-3.0 mmol L^-1^), the uptake of honokiol increased slowly, as shown in **Figure 4A**. The uptake was consistent with the enzymatic kinetic model, and the kinetic parameters were Km = 0.365 mmol L^-1^ and Vmax = 0.231 μmol L^-1^ h^-1^. These results indicate that the uptake of honokiol by PCH is a biochemical process, and the uptake process involves the carrier. The results of our study are consistent with the previous study, which reported that uptake of *Ricinus communis* to glucose-fipronil conjugate is in accordance with the Michaelis-Menten equation, which is an active uptake process (Mao et al., 2017). Transport study has indicated that some organic solute transport modes are mediated by the simultaneous operation of saturable and non-saturable uptake (Conde et al., 2006).

### 3.5 Effect of temperature on the uptake of foliar applied honokiol by PCH

Under 24 °C and 4 °C temperature treatments for 24 h, the amounts of honokiol in the stem of PCH were found to be 0.887 ± 0.0019 mg kg^-1^ and 0.2012 ± 0.0011 mg kg^-1^, respectively, as shown in **Figure 4B**. The contents of honokiol at two different temperature treatments increased gradually with the increase of culture time, and the contents of chemicals at each culture time at 24°C were higher than those at 4°C. The value of Q_10_ increased gradually over time, with the lowest value being 2.09 and the highest value being 2.32, both of which were greater than 2.0. It can be concluded that the uptake of honokiol by PCH is an active process related to biological metabolism. The results of our study are consistent with the previous study, which reported that a Q_10_ value greater than or less than 2.0 is used as a criterion to determine whether the uptake of a pesticide agent by plants is related to biological metabolism or belongs to passive physical processes (Singer and McDaniel, 1982). Glucosinolate uptake by leaf protoplasts of *Brassica napus* and the average temperature coefficient (Q_10_) determined from the data obtained between temperatures ranging from 4 to 30°C was 1.8 ± 0.2, which indicates that active processes might be involved (Chen and Halkier, 2000).

### 3.6 Effect of pH on the uptake of foliar applied honokiol by PCH

The applied pH treatments (4-9) showed variation in the amount of honokiol uptake by the root, stem and leaves of PCH, as shown in **Figure 4C**. In the case of the root, the highest content of honokiol was found to be 1.0462 ± 0.0057 mg kg^-1^ when pH = 4.0; and at pH 7.0, the content was 0.2074 ± 0.0063 mg kg^-1^, indicating that the content of honokiol at pH = 4.0 was five times higher than that at pH = 7.0. For the stem, honokiol content was found to be highest at pH = 6, which was 0.9145 ± 0.0068 mg kg^-1^, and at pH 8.0, the content was 0.3066 ± 0.0059 mg kg^-1^, indicating that the content of honokiol at pH = 6.0 was twice as much as that at pH = 8.0. For leaves, honokiol content was found to be highest at pH = 5.0, which was 2.4508 ± 0.0016 mg kg^-1^, and at pH 9.0, the content was 0.4419 ± 0.0017 mg kg^-1^, indicating that the content of honokiol at pH 5.0 was five times higher than that at pH 9.0.

The findings showed that application of different pH had a significant effect on the uptake of honokiol by PCH, and it can be said that the uptake of honokiol in PCH was more favorable under weak acid conditions. The intracellular proton concentration was affected by the external pH value, and the uptake of honokiol by PCH was affected by pH change, indicating that the uptake of honokiol by PCH was driven by the concentrations of protons in the plasma membrane of PCH. The results of our study are consistent with the previous study which reported that uptake of imazethapyr and imidazolinone in plants is affected by solution pH and the uptake of glucosinolate by leaf protoplasts of *B. napus* is affected by pH solution (Chen and Halkier, 2000; Spackman and Cobb, 2000).

### 3.7 Effect of energy inhibitors on the uptake of foliar applied honokiol by PCH

**Figure 5:**
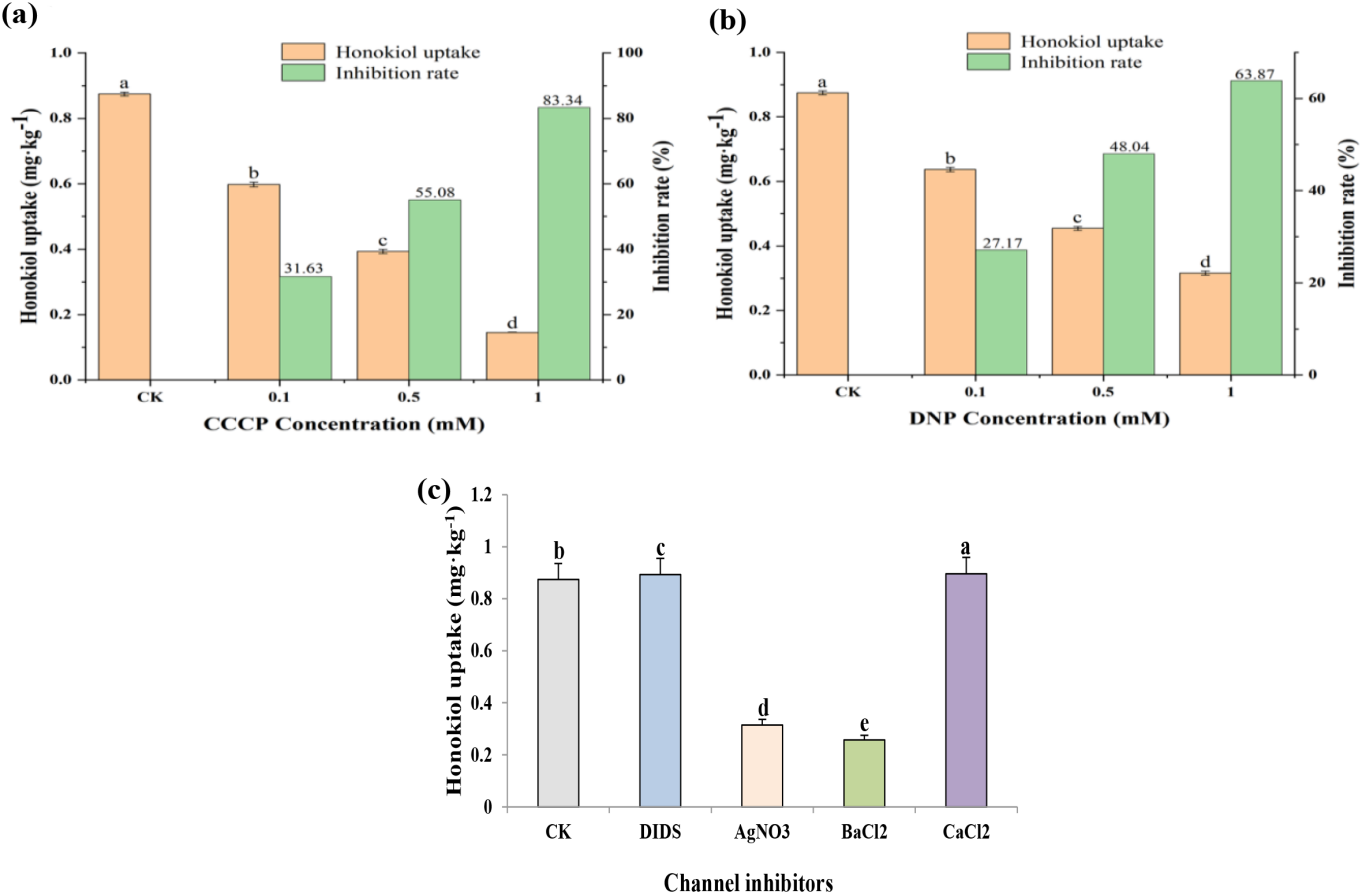
**(a, b)** Impact of energy inhibitors (CCCP & DNP) on the uptake of honokiol in PCH. **(c)** Effect of specific channels inhibitors on the uptake and transport of honokiol in PCH. The bars sharing the same letters are not significantly different from each other, while the bars with different letters are significantly different (P<0.05).

In order to further verify whether the uptake of honokiol by PCH requires energy consumption, energy inhibitors CCCP and DNP were used for verification. The content of honokiol in the stem of PCH was found to be 0.8748 ± 0.0061 mg kg^-1^ in the control group. After the application of different concentrations of energy inhibitors, viz., DNP and CCCP, the uptake of honokiol was significantly inhibited, and the content of honokiol decreased with the increase in the amount of inhibitor. After CCCP treatment, the honokiol contents were found to be 0.5982 ± 0.007, 0.393 ± 0.0065 and 0.1457 ± 0.0007 mg kg^-1^, and the inhibition rates were 31.63%, 55.08% and 83.34%, respectively (**Figure 5A**). After DNP treatment, the honokiol content was 0.6371 ± 0.0068, 0.4545 ± 0.0062 and 0.3161 ± 0.0066 mg kg^−1^, and the inhibition rates were 27.17%, 48.04% and 63.87%, respectively (**Figure 5B**). It was found that the process of honokiol entering PCH is an energy-consuming process. The results of our study align with previous research indicating that DNP inhibits energy production in cells, while CCCP, an uncoupling agent, affects the uptake of compounds by plants and eliminates the driving force of protons in the trans-membrane process, which is a key criterion for determining whether energy is needed during the uptake process (Ludwig et al., 2000).

### 3.8 Effect of channel inhibitors on the uptake of foliar applied honokiol by PCH

The effects of various channel inhibitors on the uptake of honokiol by PCH were shown in **Figure 5C**. The uptake of honokiol was significantly inhibited after the treatment with an aquaporin inhibitor (AgNO_3_), and the content of honokiol was found to be 0.3146 ± 0.0064 mg kg^-1^. There was no significant effect found on the uptake of honokiol after treatment with anion channel inhibitors (DIDS), and the content of honokiol was 0.8927 ± 0.0077 mg kg^-1^. After potassium ion channel inhibitor (BaCl_2_) treatment, honokiol uptake was significantly inhibited, and the content of honokiol was found to be 0.2573 ± 0.0043 mg kg^-1^. The uptake of honokiol in PCH was not significantly affected by calcium ion channel inhibitor (CaCl_2_) treatment, and the content of honokiol was found to be 0.8961 ± 0.0056 mg kg^-1^.

The results indicated that the uptake of honokiol in PCH was a process involving water channels and potassium ion channels. Our results were consistent with other findings, which reported that aquaporin inhibitors (AgNO_3_) had no significant effect on the uptake of oxytetracycline by maize and cyetpyrafen by rice and lettuce, and aquaporin may not participate in the uptake of oxytetracycline and cyetpyrafen (Kong et al., 2007). DIDS was used to verify the uptake of di-n-butyl phthalate (DNBP) by Chinese cabbage, and it was found that the process did not involve anion channels (Cheng et al., 2021). Calcium channel inhibitors can inhibit germination or significantly cause pollen tube transformation (Zhang et al., 2007). The secretion of potassium ions is inhibited by potassium ion inhibitors; the organic acids secreted by root tips under aluminum stress are also hindered (Kataoka et al., 2002).

### 3.9 UPLC-MS/MS profiling (mass spectrometry conditions & chromatographic conditions)

**Figure 6:**
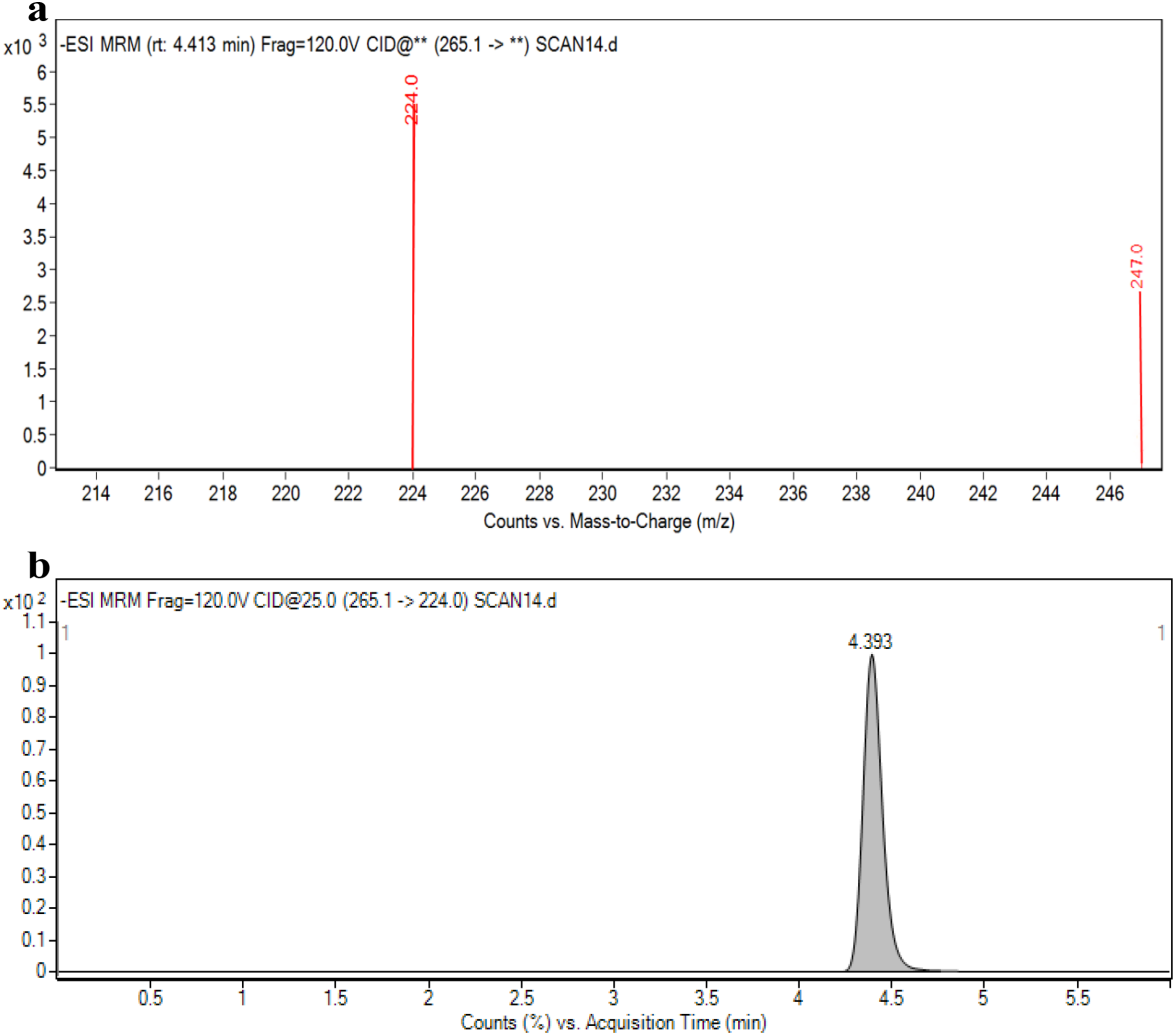
**(a)** Most abundant mass-to-charge ratio of honokiol within the time period of 4.393 ± 0.05 min was 265.1 m/z. Where “**” indicates that full scan of all fragment ions rather than a specific monitored transition. **(b)** Honokiol was completely separated with no tailing under isocratic elution using at retention time 4.393 min.

Based on the molecular structure of honokiol, a standard solution (1 mg L^-1^) was subjected to primary mass spectrometry analysis in negative ESI mode. The precursor ion, fragmentor voltage, product ion, and collision energy in MRM mode were manually optimized accordingly. As shown in **Figure 6A**, the most abundant mass-to-charge ratio of honokiol within the time period of 4.393 ± 0.05 min was 265.1 m/z. Therefore, m/z 265.1/224 was selected as the quantification ion pair with a collision energy of 25 eV, and m/z 265.1/247 was selected as the qualification ion pair with a collision energy of 35 eV. The fragmentor voltage was set at 120 V. Honokiol at 1 mg L^-1^ standard was used to compare the response intensity of isocratic and gradient elution. As shown in **Figure 6B**, honokiol was completely separated with no tailing under isocratic elution using an acetonitrile-0.1% formic acid solution (v/v) as the mobile phase, resulting in excellent chromatographic separation. Therefore, this gradient condition was selected for detection. The retention time (RT) of honokiol was 4.393 min.

### 3.10 Molecular docking analysis

**Figure 7:**
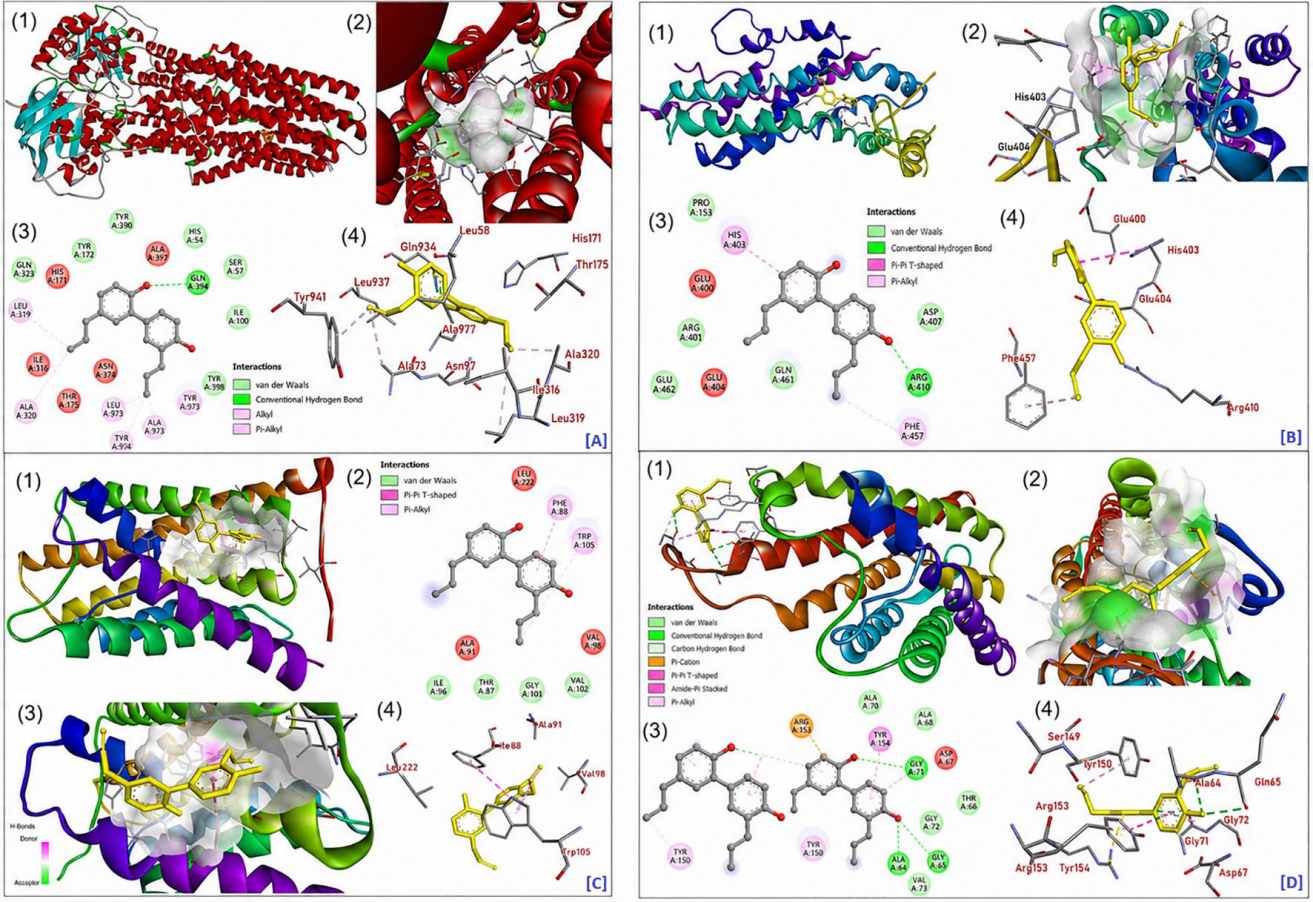
Molecular docking analysis of honokiol with transporter proteins of *A. thaliana*. **(A1-A4)** Docking interactions with ABCB11, showing the overall docked conformation, binding pocket surface, 2D interaction map, and key interacting residues. **(B1-B4)** Docking interactions with ABCG24. **(C1-C4)** Docking interactions with TIP1-2. **(D1-D4)** Docking interactions with PIP1-3. Hydrophobic, π–π, and hydrogen-bond interactions are highlighted.

Molecular docking studies were conducted to explore the interaction of honokiol with key membrane transporters of *A. thaliana*, including ABC transporters (ABCB11 and ABCG24) and aquaporins (TIP1–2 and PIP1-3). The results revealed strong and specific binding interactions, suggesting that honokiol may utilize multiple transport pathways within plant tissues. Among the tested proteins, honokiol showed the highest affinity for ABCB11 (-12.83 kcal·mol^-1^). The ligand was deeply embedded within the transmembrane substrate-binding cavity, forming stabilizing hydrophobic and π-alkyl interactions with residues Ala320, Leu319, Leu937, Ala973, and Tyr941 [**Figure 7A** (1–4)]. These interactions are typical of lipophilic substrates recognized by ABC transporters, indicating that ABCB11 may play a significant role in honokiol’s cellular trafficking and long-distance transport.

Honokiol also bound favorably to ABCG24 (−10.70 kcal·mol^-1^), positioned within the central translocation channel. The π-π T-shaped interaction with His403 and π-alkyl contact with Phe457, supported by nearby Glu400, Glu404, and Arg410 residues, stabilized the complex [**Figure 7B** (1–4). The interaction pattern suggests that honokiol’s aromatic scaffold fits well within the hydrophobic cavity of ABCG24, a transporter known for phytohormone export, implying its potential role in honokiol mobilization across membranes. Docking with aquaporins revealed moderate yet stable affinities. For TIP1-2 (-9.38 kcal·mol^-1^), honokiol interacted mainly through π-π and π-alkyl contacts with Phe88 and Trp105, aided by hydrophobic residues Ala91, Val98, and Leu222 [**Figure 7C** (1–4)]. Such interactions suggest a possible modulatory role of honokiol within the channel pore, facilitating its redistribution between vacuolar and cytoplasmic compartments. With PIP1-3 (-10.67 kcal·mol^-1^), honokiol occupied the pore-lining region, stabilized by a hydrogen bond with Gln65, a π-cation interaction with Arg153, π–π stacking with Tyr154, and hydrophobic contacts with Tyr150, Gly71, and Gly72 [**Figure 7D** (1–4)]. These specific interactions indicate that PIP1–3 could assist honokiol passage across the plasma membrane, contributing to its cellular uptake and systemic translocation.

Overall, honokiol displayed strong affinities toward both ABC transporters and aquaporins, primarily driven by hydrophobic, π-π, and hydrogen-bonding interactions (**Table 3**). These findings suggest a cooperative transport mechanism wherein ABCB11 and ABCG24 mediate long-distance movement, while TIP1–2 and PIP1–3 facilitate intracellular exchange. Such dual transport pathways likely enhance honokiol’s mobility and bioavailability within *A. thaliana*, providing a mechanistic basis for its efficient distribution and physiological activity. The idea that honokiol can directly interact with the transporter protein and possibly alter its activity is supported by the docking scores and binding free energy values. The ABC transporters are one of the largest and most diverse protein families; they use adenosine triphosphate (ATP) hydrolysis to drive the efflux or influx of metabolites, xenobiotics, hormones, and signaling molecules and control the movement of a wide range of molecules across biological membranes (Higgins, 2007; Wilkens, 2015). These insights not only improve our understanding of honokiol’s cellular transport mechanisms but also open up new perspectives for optimizing its delivery and utilization in both agricultural and therapeutic contexts.

**Table 3:** Types of bonds involved in the interaction of ABCB11, ABCG24, TIP1–2 and PIP1–3 with honokiol.

S.N.	Amino acid involved	Atoms of honokiol	Type of interactions involved	Distance (Å)
Types of bonds involved in the interaction of ABCB11 with honokiol				
1	ALA320	C20	Alkyl	3.59154
2	ALA973	C19	Alkyl	4.35633
3	LEU937	C19	Alkyl	5.36034
4	LEU319	C20	Alkyl	5.48952
5	TYR941	C19	Pi-Alkyl	4.46064
Types of bonds involved in the interaction of ABCG24 with honokiol				
1	HIS403	Benzene Ring	Pi-Pi T-shaped	4.72856
2	PHE457	C19	Pi-Alkyl	4.45443
Types of bonds involved in the interaction of TIP1–2 with honokiol				
1	PHE88	Benzene Ring	Pi-Pi T-shaped	4.99985
2	TRP105	C19	Pi-Alkyl	5.00219
Types of bonds involved in the interaction of PIP1–3 with honokiol				
1	GLN65	O_2_	Conventional Hydrogen Bond	3.2699
2	ARG153	Benzene Ring	Pi-Cation	3.67721
3	TYR154	Benzene Ring	Pi-Pi T-shaped	5.56737
4	GLY71	Benzene Ring	Amide-Pi Stacked	3.48279
5	GLY72	Benzene Ring	Amide-Pi Stacked	3.48279
6	TYR150	C20	Pi-Alkyl	4.19342

At present, chemical agents are often used to control the root rot disease of PCH. However, the long-term and widespread use of chemical pesticides with the same mechanism of action is likely to lead to 3R (resistance, residue, and rampant) problems, which can pollute the environment and put people’s health at risk (Su et al., 2023). Therefore, it is particularly important to discover new ways to control plant diseases with low toxicity and high efficiency. Honokiol showed higher antifungal activity against *R. solani* than tebuconazole and also reported that honokiol could lead to excessive production of reactive oxygen species (ROS), which disrupts mitochondrial function, affects respiration, blocks the tricarboxylic acid (TCA) cycle, and ultimately inhibits ATP production (Yan et al., 2020). Fungicide application strategies include but are not limited to diverse techniques such as foliar sprays, soil applications, fumigation treatments, and seed treatments (Kabdwal et al., 2019; Sahoo and Kadoo, 2024). Among these methods, the conventional root irrigation method requires excessive use of pesticides due to soil adsorption, which is easy to cause economic losses and environmental pollution, and it is time-consuming and laborious. Therefore, foliar spraying has become the main means of crop protection in the field due to its convenience and efficiency. At present, most of the systemic pesticides are mainly transported to the top of the plant through the xylem and driven by transpiration flow (Zhang and Li, 2023). Pesticides that can be transported via phloem are of great significance for the prevention and treatment of root diseases, vascular bundle diseases and piercing-sucking pests. However, only a few pesticides can be transmitted downward through the phloem of plants. Among them, phenamacril has attracted attention because of its excellent phloem transmission ability from bud to root (Li et al., 2023).

Our study exclusively explored the uptake and transport rules of honokiol in PCH through foliar spraying. The study initially revealed the conduction characteristics of honokiol in PCH can be absorbed through the leaves and effectively transmitted to the roots through the symplastic pathway in the phloem. However, there is still no clear evidence on whether honokiol can be absorbed by roots and transported upward to other parts of the plant through the apoplastic pathway of xylem. In order to further explore this possibility, a hydroponic exposure experiment can be designed to observe the transport behavior of honokiol in plants by controlling the root environment. In this study, the uptake of honokiol by PCH was significantly inhibited by the water channel inhibitor, silver nitrate, and the potassium channel inhibitor, barium chloride, but not significantly correlated with anion channels and calcium channels. Honokiol has a certain internal uptake, and the bio-concentration factor (BCF) of honokiol in PCH shows obvious site specificity.

This study only clarified that the uptake and transport of honokiol in PCH is a carrier-mediated active uptake process that requires energy, but the key genes related to transmembrane transport are still unclear. In order to deeply understand the uptake and transport mechanism of honokiol in PCH, future research can use high-throughput transcriptome sequencing technology to conduct a comprehensive gene expression analysis of different parts of PCH treated with honokiol. Through this method, genes with co-expression patterns during trans-membrane transport can be identified and screened, and these genes may encode specific transporters involved in honokiol transport in phloem. Through further functional verification and bioinformatics analysis, the specific role of these candidate genes in the trans-membrane transport of honokiol can be clarified, thus offering novel insights into the molecular mechanism of compound transport in plants. In summary, exploring the uptake and transport pattern of honokiol in PCH provides a scientific basis for promoting the uptake of honokiol and improving the utilization rate of honokiol. It provides important information support for the application of such agents in the field and ensures the scientificity and rationality of the application strategy. Additionally, it established a solid theoretical foundation for future studies on targeted pesticides. In the future, we can further study how to regulate various factors, improve the transport ratio of honokiol to roots, and improve the control effect.

## 4 Conclusions

This study unequivocally shows that when foliarly administered, honokiol actively translocates basipetally down the phloem stream rather than remaining restricted to aerial tissues. The substance travels in a certain direction from leaves to stems before building up in the root system, which is the site of the most intense pathogenic attack. Because honokiol can function directly in the rhizosphere and root tissues due to this focused delivery, it ensures localized antifungal activity precisely where root-rot pathogens invade. Because of its systemic basipetal mobility, honokiol offers a dual benefit over traditional foliar-applied fungicides, which only function at the application site. This advantage is because it protects the shoot while also strengthening the root zone. The promise of honokiol as a systemic plant-protection agent is highlighted by its capacity to migrate directionally from aerial parts to subterranean organs, turning a basic foliar spray into whole-plant defense. The uptake and transport mechanism and dynamic distribution of honokiol in PCH were preliminarily clarified, and it was proved that honokiol could carry out two-way conduction in PCH, mainly downward conduction. It was observed that the process of honokiol uptake by PCH was sensitive to temperature and pH and significantly inhibited at low temperature (4 °C), and it was more conducive to the uptake under weak acid conditions. Water channels and potassium channels are involved in the uptake and transport of honokiol; it can be absorbed by the leaves of PCH and transported to the roots through the phloem. The uptake and transport of honokiol in PCH is an active uptake process mediated by the carrier. Strong and consistent binding affinities with important transporter proteins like ABCB11, ABCG24, TIP1-2, and PIP1–3 demonstrate honokiol’s potential as a promising bioactive substance, according to a molecular docking study. Honokiol may function as a natural regulator of transporter activity, opening up new possibilities for its use in improving stress tolerance, controlling metabolite transport, and developing the agricultural sector. These interactions may also offer new insights into the molecular underpinnings of transport-associated mechanisms in plants. All of these results point to basipetal translocation and root accumulation of honokiol as a novel mechanism that may transform natural product-based approaches to managing root diseases in a sustainable manner.

## Data Availability

The original contributions presented in the study are included in the article/**Supplementary Material**. Further inquiries can be directed to the corresponding author/s.
