# Supplementary material for: Foliar-applied honokiol exhibits basipetal translocation, offering a strategy for root disease management in precision agriculture systems
Source: Front Plant Sci. 2026 May 21;17:1846319. doi: 10.3389/fpls.2026.1846319 (PMC13233685; doi:10.3389/fpls.2026.1846319)
Supplement: Supplementary file 1 [file DataSheet1.docx]

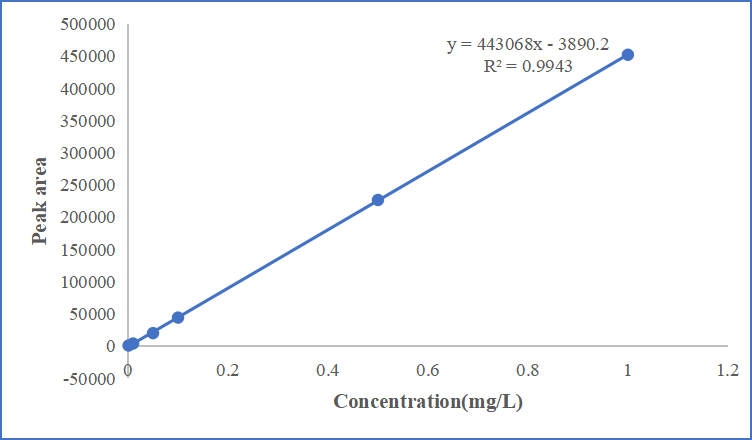


**Fig. 1** Regression equation was y=443068x-3890.2 and correlation coefficient*=* 0.9943, indicating linear relationship between concentration and corresponding peak area.

**Table 1** Recovery and relative standard deviation of honokiol at different addition levels.

| Matrix | Addition level (mg/kg) | Average recovery rate (%) | Relative standard deviation (%) |
| --- | --- | --- | --- |
| Root | 0.1 | 94.27 | 6.92 |
|  | 0.5 | 87.26 | 6.91 |
|  | 1 | 92.02 | 7.09 |
| Stem | 0.1 | 90.71 | 5.33 |
|  | 0.5 | 83.37 | 3.36 |
|  | 1 | 92.74 | 5.42 |
| Leaf | 0.1 | 89.43 | 5.09 |
|  | 0.5 | 95.43 | 5.56 |
|  | 1 | 94.18 | 2.85 |
